# The Prognostic Significance of QTc Prolongation in Lead aVR in Patients with Acute Coronary Syndrome with ST Elevation or Depression

**DOI:** 10.3390/medicina60122038

**Published:** 2024-12-11

**Authors:** Abuzer Coskun, Burak Demirci, Mehmet Oktay Alkan, Selman Gundogan, Sevki Hakan Eren

**Affiliations:** 1 Department of Emergency Medicine, Istanbul Bagcilar Training and Research Hospital, Istanbul 34200, Turkey; drburakdemirci@hotmail.com (B.D.); moktayalkan26@hotmail.com (M.O.A.); selman.gundogan26@gmail.com (S.G.); 2 Department of Emergency Medicine, Faculty of Medicine, Gaziantep University, Gaziantep 27410, Turkey; shakaneren@gmail.com

**Keywords:** acute coronary syndrome, emergency department, aVR ST elevation–depression, QTc

## Abstract

*Background and Objectives*: In patients with acute coronary syndrome, electrocardiographic parameters, including ST elevation in lead aVR (aVR-STE), ST depression (aVR-STD), and QTc prolongation, are crucial. This study aims to show the predictive value of a longer QTc in emergency department patients with acute coronary syndrome and ≥1 mm ST elevation or depression in the aVR lead in electrocardiography. *Materials and Methods*: A retrospective analysis was conducted on 1273 patients admitted to the emergency department with a preliminary diagnosis of acute coronary syndrome between 2020 and 2023. ST depression, ST elevation, and QTc were documented in the electrocardiography of the patients. Furthermore, acute coronary syndrome subtypes were identified. Basic demographic characteristics, complications, concomitant diseases, and 30-day and 180-day mortality data were collected. *Results*: The mean age of 1273 patients included in the study was 63.23 (10.06) years and 548 (43%) were female (*p* = 0.030). In the aVR-STE group, the QTc was 483.31 (33.96) ms in STEMI, 474.98 (26.21) ms in NSTEMI, and 505.60 (9.76) ms in those with mortality (*p* < 0.001). In the aVR-STD group, the QTc was 465.10 (42.63) ms in STEMI, 457.52 (39.52) ms in NSTEMI, and 508.73 (4.71) ms in those with mortality (*p* < 0.001). The total 30-day mortality was 129 (10.1%) and 180-day mortality was 181 (14.2%) (*p* < 0.001). In the uni-multivariable regression analysis performed for both change in aVR derivation and mortality, it was determined that prolonging QTc could be a predictive value for acute coronary syndrome (*p* < 0.001). We found sensitivity at 99.7% and specificity at 99.2% in predicting mortality in patients with prolonged QTc (AUC: 0.983, 95% CI: 0.974–0.993, *p* < 0.001). *Conclusions*: In patients with acute coronary syndrome, a prolonged QTc is an independent predictor of short- and long-term mortality in alterations in aVR derivation.

## 1. Introduction

Worldwide, acute coronary syndromes are a major problem for public health. Additionally, they cause substantial morbidity and mortality among the general population [1]. Acute coronary syndrome falls into three categories: ST elevation myocardial infarction (STEMI), non ST elevation myocardial infarction (NSTEMI), and unstable angina. They also comprise a large group of people who may or may not show changes in their electrocardiograms, sudden rises in cardiac troponins, or other symptoms [2]. The incidence of ST elevation myocardial infarction in acute coronary syndromes varies from 29% to 47% [3]. Non ST elevation myocardial infarction causes approximately two-thirds of all myocardial infarctions [4]. Acute coronary syndrome results in approximately 2.2 million (38%) deaths in women and 1.9 million (44%) deaths in men each year [5].

Acute coronary syndrome clinical practice relies heavily on the electrocardiogram for diagnosis, risk classification, and treatment response monitoring [6]. The 12-lead electrocardiogram includes the augmented voltage right (aVR) as one of its unipolar leads. You can use the aVR lead to gain insight into the basal portion of the cardiac septum and the outflow tract of the right ventricle. To put it another way, the aVR is located opposite the lateral wall of the left ventricular cavity and the interior part of the apex. Therefore, it opposes I, II, V5, and V6 [7,8]. The 2013 American Heart Association STEMI management guideline [9] indicates that aVR ST elevation (aVR-STE) accompanied by ST depression in multiple leads is associated with left main or proximal left descending coronary artery occlusion. According to the 2017 European Society of Cardiology guideline on the management of patients with ST elevation myocardial infarction, the presence of hemodynamic deterioration, and an ST segment depression of 1 mm or more in “8 or more” surface leads, accompanied by ST segment elevation in “aVR and/or V1”, may indicate multivessel ischemia and left main coronary artery obstruction [10]. In 2018, the fourth universal definition of myocardial infarction emerged, recognizing aVR-STE in combination with specific repolarization patterns as the equivalent of ST elevation myocardial infarction [11]. In addition, an ST segment depression of 1 mm or more in 6 leads and ST segment elevation in aVR or V1, accompanied by “hemodynamic deterioration”, is defined as a multivessel disease or left main coronary artery disease [11].

Numerous studies have examined the relationship between electrocardiographic (ECG) and angiographic findings and the outcomes of these patients. These studies have associated ST segment changes in the aVR lead with poor outcomes in patients with in-hospital MI [12,13]. In a study by Kukla et al., patients with ST elevation in lead aVR were 1.5 times more likely to have neurological deterioration than those with ST segment sparing. They were also approximately 30 times more likely to have a fracture than those without ST segment changes [14]. Although ST segment depression in lead aVR is difficult to diagnose, it can be a good indication of ischemia or injury in the apex and inferolateral regions of the heart. The presence of ST segment depression indicates that a larger area of the myocardium is affected and may indicate the need for more invasive interventions [15,16]. It has been shown that having an ST elevation in the aVR lead increases the risk of having another myocardial infarction, heart failure, and the need for coronary artery bypass surgery [15]. There was a study by Kosuge et al. that found that an ST segment elevation of more than 0.05 mV in lead aVR could accurately predict the involvement of all three heart vessels 78% of the time [17].

The QT interval is the time from the beginning of the Q wave to the end of the T wave on the electrocardiogram and is a noninvasive biomarker of ventricular repolarization. An abnormally prolonged QT interval (>450 ms in men and >460 ms in women) can lead to early post-depolarizations and early action potentials, ventricular arrhythmias, and sudden cardiac death [18]. This underlies the persistent changes in corrected QT (QTc) observed in patients with acute myocardial infarction. Studies have shown that QTc prolongation precedes ST segment elevation at the onset of transmural ischemia in patients with acute myocardial infarction [19]. The ACTION study showed that a QTc longer than 430 ms was predictive of death in patients with coronary artery disease [20]. Long QTc levels were found in people with unstable angina or an acute myocardial infarction, but they went back to normal 48 h after successful myocardial revascularization [21].

This study aims to show how useful a prolonged QTc is for predicting what will happen in people who go to the emergency room with an acute coronary syndrome diagnosis and an aVR lead that is ≥1 mm ST elevated or depressed on an electrocardiogram.

## 2. Materials and Method

***Study design and population:*** This retrospective study was conducted on patients admitted due to acute coronary syndrome at the Emergency Medicine Clinic of the Health Sciences University, Bağcılar Training and Research Hospital in Istanbul, Turkey, between 1 January 2020 and 31 December 2023. The study encompasses 1273 patients aged 18 and above admitted to the emergency department for acute coronary syndrome and diagnosed with ST elevation and depression in lead aVR. Our hospital’s registration system includes the patients’ diagnoses, admission dates, contact information, and demographic, clinical, and laboratory data. The study excluded 28,378 cases, where patients with aVR derivation on the isoelectric line and stable angina diagnosis underwent 30-day and 180-day follow-ups using the ‘e-pulse’ and automation systems. Thus, it was possible to compare the groups with and without changes in aVR derivation.

***Data collection:*** We classified the patients into two groups, aVR ST elevation and aVR-ST depression, based on changes in aVR derivation. We created three groups based on the patients’ acute coronary syndrome status: unstable angina, ST elevation myocardial infarction, and NSTEMI. We also recorded the two- and three-vessel diseases, complications, and the mortality status of the cases. We also recorded the comorbidity status of the patient population. When these patients applied to the emergency department, we obtained their age, gender, blood sugar, lipid profile, hemogram, and blood samples for biochemistry from their records and patient files. We also evaluated their electrocardiography and QTc values from the automation system. The automation system also checked patients’ reapplications within an average of 180 days after the follow-up. The results of the cases were to other hospitals either via our country’s “electronic health data” system or by telephone.

In all cases included and excluded from the study, it was required not to have a COVID-19 infection or to have a negative polymerase chain reaction test. Therefore, we evaluated a more homogeneous patient population.

***Inclusion and exclusion criteria:*** The study included patients admitted to our Emergency Medicine Clinic diagnosed with acute coronary syndrome from 2020 to 2023. Patients diagnosed with high-risk unstable angina, ST elevation myocardial infarction, and NSTEMI, exhibiting aVR ST elevation and aVR ST depression in the aVR lead on electrocardiography, were included in the study. Two impartial evaluators conducted a blind assessment of the electrocardiograms acquired upon admission. Measurements were conducted using both the monitoring system and a printed ruler manually. In instances of interpretation divergence, consensus was achieved through deliberation among the raters. ST segment elevation and depression in lead aVR were assessed from the J point. In lead aVR, ST elevation was defined as a height of ≥1 mm in at least two contiguous leads, whereas ST depression was defined as a decrease of ≤1 mm. Individuals aged 18 and older who satisfied these criteria were incorporated into the study. Our exclusion criteria included patients under 18 years of age, individuals with missing laboratory results and registration data, those exhibiting normal ST segments in an aVR derivation, and patients whose blood glucose levels were not assessed during the initial 24 h. Furthermore, cerebrovascular diseases, mental disorders, chronic liver diseases, dialysis resulting from chronic renal failure, chronic inflammatory conditions, malignancies, and hematological disorders were excluded from the study. Moreover, numerous causes extend QTc duration. Individuals with hypokalemia, hypomagnesemia, hypocalcemia, post-cardiac arrest, elevated intracranial pressure, congenital long QT syndrome, and certain drug usage were excluded from the study.

***Definitions:*** ST elevation myocardial infarction—the evaluation of patients exhibiting acute chest pain should begin with an ECG and troponin assays. The American College of Cardiology (ACC), American Heart Association (AHA), European Society of Cardiology (ESC), and World Heart Federation (WHF) committee established the following ECG criteria for ST elevation myocardial infarction (STEMI): New ST segment elevation appears at the J point in two adjacent leads, surpassing a threshold of 0.1 mV in all leads except V2 and V3. The threshold in leads V2 and V3 surpasses 0.2 mV for males over 40, 0.25 mV for men under 40, and 0.15 mV for women [11]. The Agency for Health Care Policy and Research guidelines on “Unstable Angina: Diagnosis and Management” classify low-risk unstable angina patients as those who do not have rest or nocturnal angina and exhibit normal or unchanging electrocardiograms. High-risk patients were defined as those exhibiting pulmonary edema; persistent rest discomfort beyond 20 min; angina accompanied by S3 gallop, rales, or a new or exacerbated mitral regurgitation murmur; hypotension; or dynamic ST segment alterations of ≥1 mm. In recommendations, unstable angina (UA) and non ST elevation myocardial infarction (NSTEMI) are closely associated conditions characterized by analogous etiology and clinical manifestations; however, they differ in severity. They are regarded as associated conditions, specifically concerning the severity of ischemia to myocardial damage and the detection of myocardial damage markers, predominantly troponin I (TnI), troponin T (TnT), or the MB isoenzyme of creatine phosphokinase (CK-MB), which varies in the quantity released. In the absence of a detectable biochemical marker for myocardial necrosis, a patient with acute coronary syndrome (ACS) may be classified as having unstable angina (UA); non ST elevation myocardial infarction (NSTEMI) is identified by the presence of the myocardial damage marker [22]. Despite several metrics being utilized in numerous studies for ST elevation and depression in lead aVR, Wong et al. [21] were selected as a reference in our research. Consequently, elevation and depression in lead aVR were defined as 0.1 mm or greater. Hypertension during office measurements or antihypertensive treatment was defined as a blood pressure of 140/90 mm Hg or higher on more than two occasions while taking in account diabetes mellitus. Diabetes mellitus was defined as a fasting blood sugar of 126 mg/dL or higher or the use of antidiabetic therapy. HbA1c is not used to define diabetes in the emergency department. Two-vessel disease or three-vessel disease of the right coronary artery is characterized by above 50% stenosis in the left coronary artery or circumflex arteries.

***Laboratory results:*** Venous blood samples were collected from the antecubital veins of patients upon their admission to the emergency room to assess blood sugar, lipid profile, troponin, hemogram, and biochemical serum levels. Troponin I levels were measured using the Troponin I STATE lecsys and Cobas e411 Hitachi Roche analyzers, Roche Diagnostics GmbH, Mannheim, Germany (Troponin I range: 0–0.05 ng/mL).

***Electrocardiography and QTc calculation:*** The patients’ electrocardiograms were obtained at the bedside using the Cardiofax electrocardiography-9132K (Nihon Kohden, Tokyo, Japan) in a 12-lead format. Patients presenting to the emergency department with a diagnosis of acute coronary syndrome were documented at a paper speed of 25 mm/s and a calibration of 1 mV/10 mm using a standard 12-lead electrocardiography apparatus upon admission. After the recording, it was uploaded to the automation system. The QT interval associated with ventricular systole was quantified from the onset of the QRS complex to the conclusion of the T wave for calibration purposes. In the context of a broad QRS (>0.12 s), a heightened upper limit of normal (500 ms) for QTc should be used [23]. Consequently, patients with a QRS duration of up to 0.12 s were included, whilst those with a QRS duration exceeding 0.12 s were excluded from the study. The QT interval must be evaluated in many leads, and the maximum QT interval recorded in any lead should be utilized. The most commonly utilized leads are first II and subsequently V5 [23]. Consequently, lead II was initially employed in the investigation; however, in instances when measurements were unattainable, such as with markedly flat T waves, lead V5 was favored. QT and RR intervals were assessed by two seasoned specialist physicians utilizing five consecutive beats in lead II. The measurement was conducted using both the automated system and manual methods. In instances of discord, the mean of both values was calculated. Measurements included significant U waves (>1 mm) that merged with the T wave or produced bifid T waves. Nonetheless, little U waves (<25% of the T wave) or distinct noticeable U waves apart from the T wave were disregarded. Given that the QT interval duration fluctuates with heart rate, the study encompassed heart rates ranging from 60 to 100 beats per minute [23]. Numerous investigations indicated the comparable detection of T and U waves. Consequently, in our investigation, the QTc was computed using the methodology established by Kotsialou et al. [23] as a reference. The investigation included 7 patients exhibiting U waves, while the numerical values of the previous ones remained unchanged. Thirteen patients exhibited a heart rate exceeding 140 beats per minute, whereas four patients demonstrated a heart rate below 60 beats per minute. The specialists who reviewed them believed they did not influence the statistical data, so they were not eliminated from the study. Since patients with heart rates outside these values were hospitalized in the services, those whose heart rates returned to normal in the first 24 h were included in the study. Therefore, the QT interval (QTc) corrected according to heart rate was calculated. The most commonly used method for QTc calculation is the Bazett formula (QTc = QT/RR^1/2^) [24]. Normally, the QTc is >360 ms, and a QTc of >450 ms in men and >460 ms in women was considered prolonged [18]. Zhang et al. [25] reported that 10–20% of healthy individuals may have QTc values outside this range. However, Moric et al. [26] reported that 2.5% of the healthy population has a prolonged QT interval.

***Ethical consideration:*** This study received approval from the Health Sciences University Bagcilar Training and Research Hospital Non-Interventional Clinical Research Ethics Committee on 22 March 2024, Decision No: 2024/03/07/031. Participants were guaranteed that their personal information, contact details, and identity data would remain confidential and undisclosed. All phases of this investigation were conducted by the stipulations of the Helsinki Declaration on Research Projects.

### Statistical Analysis

During the indicated study period, 29,651 individuals presented to the emergency department with chest pain. The study comprised instances of high-risk unstable angina, ST elevation myocardial infarction, and non ST elevation myocardial infarction. Patients with stable angina, those with a normal ST segment in the aVR derivation, and individuals experiencing chest discomfort unrelated to coronary artery disease were excluded from the trial and monitored only for death outcomes. Patients with acute coronary syndrome were incorporated into the G*Power study based on ST segment alterations in the aVR lead. The analysis conducted using G*Power [27] included 1273 patients, derived from a population of 29,651 cases, with a 5% acceptable error margin and a 95% confidence range. Cases excluded from the trial were monitored for control purposes via the automation system for mortality at 30 and 180 days post application. The objective was to ascertain the mortality rate among populations with and without aVR changes.

The data acquired from this investigation were analyzed using the SPSS 26.0 software package (SPSS Inc., Chicago, IL, USA). The One-Sample Kolmogorov–Smirnov test was employed to assess if the variables originated from a normal distribution. The Kruskal–Wallis H Test and the Mann–Whitney U Test were employed to analyze the differences between the groups, as the variables, except the QTc, did not follow a normal distribution. The Student *t*-test was employed for QTc distance, while the Duncan test was utilized for intergroup comparisons. Duncan’s multiple comparison analysis was employed to evaluate ST elevation and depression in lead aVR, acute coronary syndrome subgroups (unstable angina, STEMI, and NSTEMI), and death rates. In the Duncan test, the initial stage for group comparison involved ranking the means from smallest to largest. Consequently, the disparities between the means were organized based on their positions in the standardized ranking. Chi-square analysis was utilized to investigate the associations among groups of nominal variables. Pearson correlation was conducted for QTc distance, while Spearman’s Rho analysis was utilized for the remaining parameters to ascertain the correlation between the groups. The covariance values of the two variables were computed for the groups with and without mortality concerning age. The correlation coefficient was determined by dividing this value by the standard deviation of the two variables. Consequently, data were provided regarding the extent of the correlation between age and the two variables. Kaplan–Meier Survival analysis was calculated for ST elevation and depression in the aVR lead. Furthermore, univariate and multivariable analyses of factors were conducted using binary logistic regression. Variables identified as statistically significant in the univariate binary logistic regression analysis were incorporated into the multivariable regression model following the forward stepwise selection approach to ascertain independent predictive factors for aVR derivation and mortality outcomes. In binary logistic regression analysis, the association between variables was deemed statistically significant at *p* < 0.01. Values above *p* > 0.01 suggested a random association, while values surpassing *p* > 0.05 were considered unimportant. Receiver operating characteristic (ROC) curve analysis was utilized to assess the sensitivity and specificity of the QTc value change in the aVR derivation concerning mortality. In the analysis of data, *p* < 0.01 for binary logistic regression and *p* < 0.05 for other variables were deemed statistically significant.

## 3. Results

The mean age of the 1273 patients included in the study was 63.23 (10.06) years, 548 (43%) were female, and the minimum–maximum age distribution range was 34–88 years. The analysis of age by ST elevation and depression groups in the aVR lead was statistically significant (*p* = 0.030). Troponin-I was 1.41 (1.89) ng/mL and mean blood sugar was 132.01 (38.87) mg/dL (*p* < 0.001) at the time of admission. Diabetes mellitus was present in 561 (44.1%), hypertension in 859 (67.5%), and tobacco use in 633 (49.7%) patients. Unstable angina was detected in 622 (48.9%) cases, STEMI in 409 (32.1%) cases, and NSTEMI in 242 (19%) cases (*p* = 0.002). Additionally, angiography revealed two-vessel disease in 234 (18.4%) cases and three-vessel disease in 158 (12.4%) cases. Both two- and three-vessel diseases were more common in the aVR-STE group (*p* = 0.001). The 30-day mortality of the patients was 103 (8.1%) in the aVR-STE group and 26 (2%) in the aVR-STD group. The 180-day mortality of the patients was 137 (10.8%) in the aVR-STE group and 44 (3.5%) in the aVR-STD group (*p* < 0.001, Table 1).

QTc, 30-day in-hospital mortality, and ST elevation and depression in the aVR lead were analyzed according to acute coronary syndrome subtypes. Accordingly, in the aVR-STE group, QTc distance was measured as 406.37 (40.01) ms in unstable angina, 483.31 (33.96) ms in ST elevation myocardial infarction, 474.98 (26.21) ms in non ST elevation myocardial infarction, and 505.60 (9.76) ms in mortality (*p* < 0.001). In the aVR-STD group, QTc was found to be 385.55 (40.83) ms in unstable angina, 465.10 (42.63) ms in STEMI, 457.52 (39.52) ms in NSTEMI, and 508.73 (4.71) ms in those with mortality (*p* < 0.001, Table 2).

**Figure 1:**
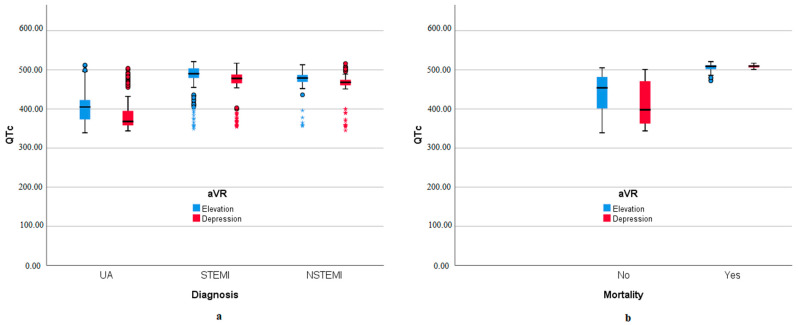
Acute coronary syndrome subtypes and mortality distribution according to QTc lead to aVR. (**a**) QTc distribution in aVR lead change according to acute coronary syndrome subtypes. (**b**) QTc distribution in aVR lead change according to mortality.

**Figure 2:**
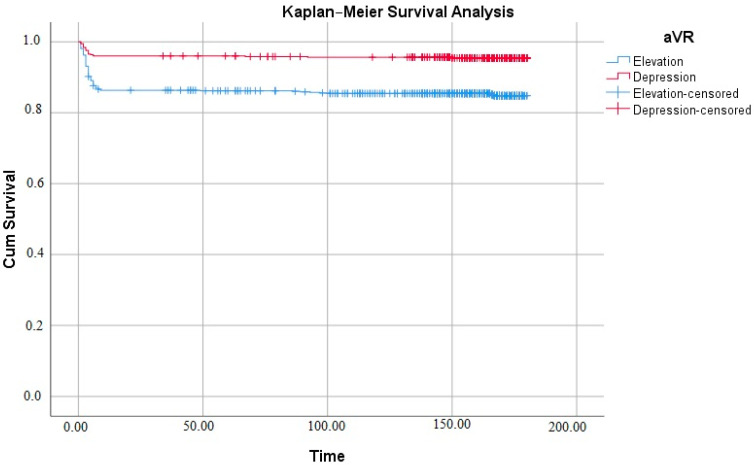
Kaplan–Meier Survival analysis.

Correlation analyses were performed with variables for aVR lead groups and mortality. For aVR groups, age, blood glucose, QTc, and troponin-I showed a negative moderate correlation. In mortality, these variables were positively and moderately correlated (Table 3). QTc distribution is given for aVR derivation in Figure 1a and for mortality in Figure 1b. In the Kaplan–Meier Survival analysis, the mean survival of the elevation group was calculated as 154.98 days [95% confidence interval (CI) 150.461–159.512] and the mean survival of the depression group was calculated as 172.53 days [95% CI 169.669–175.394], and the distribution is given in Figure 2.

Univariate and multivariable regression analysis was performed with variables for mortality and aVR groups. All variables for both mortality and aVR groups were found to be significant in univariate analysis. In the multivariable analysis of aVR groups, it was found that blood sugar, QTc, and troponin-I could be a predictive value. In the multivariable analysis performed for mortality, it was found that only QTc distance could be a predictive value for acute coronary syndrome due to ST change in aVR derivation (Table 4).

**Figure 3:**
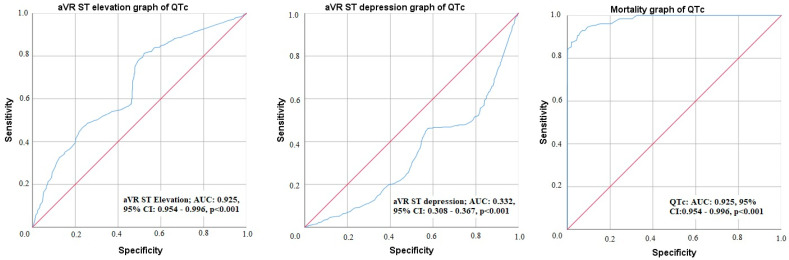
Receiver operating characteristic curve graph of aVR lead ST elevation and depression and mortality.

The receiver operating characteristic (ROC) curve analysis to predict changes in aVR derivation and mortality development is given in Figure 3. Accordingly, the sensitivity of mortality according to QTc distance was 99.7% and specificity was 99.2% [area under the curve (AUC): 0.983, 95% confidence interval (%95 CI): 0.974–0.993)]. According to the QTc distance of aVR drive, sensitivity for aVR ST elevation was 79.3% and specificity was 74.7% [AUC: 0.663, 95% CI: 0.633–0.692)]. In aVR ST depression, sensitivity was 56.7% and specificity was 53.9% [AUC: 0.337, 95% Cl: 0.308–0.367)].

**Figure 4:**
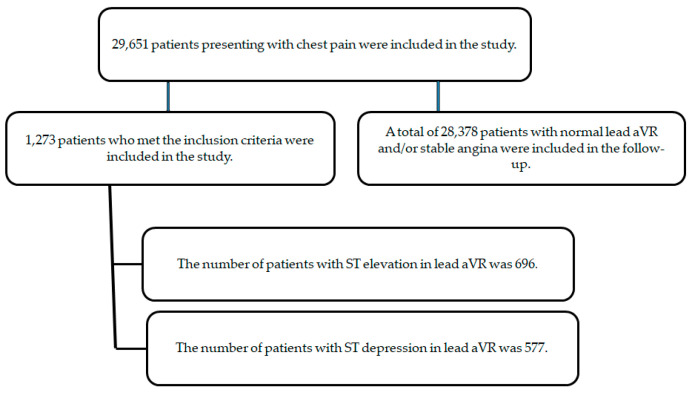
Distribution of patients in the study.

The 28,378 patients who could not be included in the study were checked by the automated system for mortality at 30 and 180 days after hospitalization. The aim was to determine the mortality rate of the QTc distance between the aVR change and the population without change. All cases resulting in mortality were identified from the records as having been admitted to the emergency department due to chest pain and subsequently hospitalized. The 30- and 180-day mortality of the 28,378 patients who could not be included in the study was checked from the automation and ‘e-nabiz’ system. In this follow-up period, the 30-day mortality of 28,378 patients, including stable angina, was determined as 64 (0.23%), and the 180-day total mortality rate was determined as 102 (0.36%). The distribution of patients in the study is given in Figure 4. To ensure the homogeneity of the study, patients diagnosed with COVID-19 or who were PCR-positive were not included in both the study group and the follow-up group.

## 4. Discussion

Multiple independent studies have examined the differences in aVR derivation and the short- and long-term correlations with QTc in acute coronary syndrome and its subgroups. The correlation between QTc prolongation and changes in aVR ST elevation and aVR ST depression among acute coronary syndrome subgroups, along with its implications for prognosis and mortality, remains undocumented in the literature. This situation led us to investigate the impact of alterations in aVR derivation on QTc prolongation in acute coronary syndrome. In all patients admitted to the emergency room with chest pain, we found that alterations in aVR derivation ≥ 1 mV and QTc prolongation in acute coronary syndromes elevated the 30-day death rate by 43.9 times and the 180-day mortality rate by 39.4 times, irrespective of age. Furthermore, our findings indicated that, in comparison to the general population and acute coronary syndrome subtypes, the elevation in QTc associated with changes in aVR derivation posed a considerable risk for two-vessel disease, three-vessel disease, and the emergence of comorbidities. Zhang et al. [28] reported a 3% prevalence of multivessel disease in people with asymptomatic cardiac disease. Adiarto et al. [29] showed a prevalence of multivessel disease ranging from 3.1% to 4.5% in patients with coronary artery disease. This work is notable as it is the inaugural study to evaluate the 30-day and 180-day mortality rates and developing comorbidities linked to the prolongation of QTc using aVR derivation.

In acute coronary syndromes, lead aVR may assist in predicting the site of the blockage of the implicated coronary artery. ST segment elevation or depression in lead aVR may yield certain implications. While the precise process remains unidentified, several possibilities have been suggested. The concurrent obstruction of blood flow in both the left circumflex and left anterior descending arteries may render the ST segment vector more perpendicular to lead V1, leading to diminished ST segment elevation in lead V1, whereas ST elevation manifests in lead aVR [30,31]. Another rationale for ST elevation in lead aVR may be that acute myocardial infarction impacts the basal septum. Lead aVR is more directed towards the basal septum compared to the other leads. The involvement of the basal septum, receiving dual circulation from both the right coronary artery and the left anterior descending artery, may result in ST elevation in lead aVR. Another idea posits that the blockage of the first septal branch or the left anterior descending artery proximal to the first septal branch may induce ST elevation in lead aVR. Nonetheless, due to the dual perfusion of the basal septum, ST elevation in lead aVR during acute anterior myocardial infarction may indicate multivessel disease that obstructs collateral perfusion or left main coronary artery disease. A correlation between ST elevation in lead aVR and multivessel or left main coronary artery disease has been documented [17,32]. Another explanation for ST elevation in lead aVR posits that there is inversion of V5 and V6 in aVR; hence, any situation inducing ST depression in V5 and V6 may result in ST elevation in lead aVR. An instance of this is anterolateral subendocardial ischemia, which may induce reciprocal ST elevation in lead aVR and ST depression in leads V5 and V6 [33]. This hypothesis posits that the blockage of the first diagonal branch may induce ST elevation in lead aVR. The spike in lead aVR has been elucidated; however, the occurrence of depression remains unexplained. Baptista et al. [34] reported that ST depression in lead aVR is not superior to traditional criteria, which include ST depression in lead I, ST depression in leads V1 and V2, ST elevation in lead III greater than II, and an ST depression in V3 to ST elevation in III ratio exceeding 1.2. The varying definitions of the infarct-related arterial, characterized as the artery exhibiting the most severe damage, may elucidate the discrepancy. The extent of ST segment depression is also significant. In patients with anterolateral myocardial infarction, ST segment depression ≥ 0.5 mm in lead aVR serves as an independent predictor of left ventricular ejection fraction < 35% before discharge, despite successful reperfusion [17]. In patients with inferior myocardial infarction, if the depression in aVR surpasses 1 mm, the likelihood of myocardial reperfusion is reduced by a factor of 8.41 [17].

*Prognostic importance of aVR derivation in acute coronary syndrome:* In the normal group, we observed aVR change at a rate of 4.3%. Of these, 45.3% were cases of aVR-STD and 54.7% were aVR-STE. In total, 7.3% to 32.3% of individuals who present with acute coronary syndrome had aVR ST elevation [35]. As a result of including patients with depression and ST elevation in aVR derivation ≥ 1 mm, we hypothesize that the low rate in our study occurs. Ghaffari et al. [36] reported a low number of patients and defined the aVR change as ≥1 mm in similar investigations. Nonetheless, there were a good number of cases, and Separham et al. [8] and Wang et al. [6] evaluated the aVR change as ≥0.05 mm and ≥0.1 mm, respectively.

The importance of lead aVR has been undervalued for numerous years. In recent years, numerous perspectives have been articulated on its significance and standing in acute coronary syndrome. Examples include the following: Lead aVR monitors electrical activity in the right ventricular outflow tract and septum of the heart. Obstruction of the left main coronary artery, impacting blood flow to the left anterior descending (LAD) artery and its branches, may result in ischemia at the base of the interventricular septum. This may lead to ST elevation in lead aVR [37]. When a substantial left ventricular region is impacted, ST-T segment depression resulting from circumflex artery (Cx) involvement is observed in aVR, occasionally in nearly all leads except V1 and III. In these leads, ST elevation manifests as a mirror pattern owing to the ischemia vector’s uphill, backward, and rightward orientation from the subepicardium to the subendocardium. Consequently, ischemia induces a negative deflection in the majority of leads [38]. Our investigation revealed that complications following acute coronary syndrome, two-vessel disease, three-vessel disease, and mortality rates were considerably elevated in both groups relative to the normal population. Moreover, these parameters were identified as independent risk factors in the aVR-STE group compared to aVR-STD. ST elevation myocardial infarction exhibited poorer outcomes compared to NSTEMI in the aVR ST elevation cohort. In a unique study, Misumida et al. [31] determined that left main coronary artery/three-vessel disease serves as an independent diagnostic criterion in STEMI patients compared to those with non ST elevation myocardial infarction, as indicated by lead aVR. Furthermore, Rathi et al. [39] illustrated in their research that the prevalence of patients with left main coronary artery or three-vessel disease in the aVR-STE group was much higher than in the aVR-STD group. A significant increase in fatalities due to ST elevation in aVR was noted.

Our data indicate the percentage of risk variables for acute coronary syndrome that remained unaltered by age as follows: 49.7% pertained to tobacco and its products, 44.1% related to diabetes, and 67.5% corresponded to hypertension. At the 30-day and 180-day follow-ups, it was seen that these factors exacerbated two-vessel disease, three-vessel disease, and death, correlating with alterations in aVR derivation. However, we could not ascertain lipid values as the cause of this increase. Investigations conducted by Ahmed et al. [7] demonstrated that significant left main coronary artery root or three-vessel disease, associated with changes in aVR derivation, was more likely correlated with diabetes, hypertension, smoking, ≥1 mm ST elevation in aVR, TIMI score ≥ 4, and left ventricular dysfunction observed via echocardiography. In Gachchhadar et al.’s study [40] on NSTEMI, over 55% of patients were smokers, 60% had hypertension, and 47.2% had diabetes. Single-vessel disease comprised 19.4% of the patients, two-vessel disease constituted 11.1%, and three-vessel disease represented 66.7%. Approximately 72.2% exhibited three-vessel disease and/or left main coronary artery involvement. In our study, 129 (10.1%) of acute coronary syndrome patients succumbed after 30 days following aVR modification, with 103 (14.7%) classified as aVR-STE cases, while 26 (4.5%) were identified as aVR-STD patients. Additionally, it was disclosed that both cohorts experienced a cumulative total of 181 (14.2%) fatalities throughout the 180-day follow-up interval. Among these, 137 (19.7%) exhibited aVR ST elevation, whereas 44 (7.6%) had aVR-STD. The 30-day mortality rate was 3.3 times greater and the 180-day mortality rate was approximately 2.6 times greater in the two groups. In a comparison with 28,378 patients admitted to the emergency department with chest pain, including those with stable angina, we observed that the 30-day mortality rate was approximately 44 times greater and the 180-day mortality rate was 39 times greater in cases of acute coronary syndrome exhibiting aVR changes. In similar studies in the normal population without aVR change, Hadanny et al. [41] reported 30-day mortality rates between 2.7% and 8% following ST elevation myocardial infarction. Nguyen et al. [42] reported the annual mortality rate after non ST elevation myocardial infarction as 3.5% and 5-year mortality as 11.4%. However, in patients with aVR change, the Prospective Global Acute Coronary Event Registry (GRACE) study revealed that in-hospital mortality was higher in NSTEMI patients with aVR-STE [43]. Wang et al. [6] reported that 90-day mortality was higher in patients with aVR ST elevation compared to patients without aVR-STE in their meta-analysis of 7700 patients. Kukla et al. [14] observed in their ST elevation myocardial infarction study on 320 people that patients with aVR ST elevation had a mortality rate of 1.5 times higher than those without aVR-STE and 30 times higher than those with aVR isoelectric line. In-hospital mortality in patients with aVR ST elevation and ST depression was five times higher compared to the standard ST elevation myocardial infarction population [44]. We believe that the correlation between QTc and ST elevation in lead aVR may soon be incorporated into the guidelines, given these findings and existing research. Consequently, we contend that LMCA and LAD could serve as robust predictors.

*Prognostic importance of QTc in acute coronary syndromes:* Alterations in QTc indicate both therapeutic measures and repolarization irregularities due to ion channel anomalies [45]. The etiology of QT prolongation can be elucidated through various mechanisms. The increase in heterogeneity often observed in ventricular myocardium cells may lead to QT prolongation [46]. Conversely, it is proposed that sympathovagal balance, primarily influenced by left ventricular systolic performance, is the principal predictor of this condition [47]. QTc markedly elevates during the acute phase in individuals with acute coronary syndrome, attributable to autonomic nervous system dysfunction and myocardial electrical disturbances resulting from cellular necrosis and electrolyte imbalances [48]. Researchers have demonstrated that the factors contributing to QT interval extension during acute myocardial ischemia include reduced the epicardial temperature, alterations in impedance, acidosis, and electrical heterogeneity of the ventricular myocardium [49]. Kenigsberg et al. [50] indicated that QTc prolongation is the initial ischemic manifestation in myocardial ischemia. The scientists determined that the molecular foundation of these observations likely resides in alterations influencing the late sodium ion current, which manifests early after myocardial ischemia and is accountable for the prolonging of the action potential.

In our study, the QTc was measured at 396 ms in the unstable angina cohort, 475 ms in the ST elevation myocardial infarction group, and 467 ms in the NSTEMI group. The QTc was measured at 447 ms in the aVR-STE group and 420 ms in the aVR-STd group. Patients with prolonged QTc had significantly greater rates of two-vessel disease, three-vessel disease, in-hospital ischemic heart failure, malignant arrhythmias, cardiac tamponade, and acute pulmonary edema compared to those without prolonged QTc. The aVR-STE group exhibited more morbidity and mortality than the aVR ST depression group, while individuals with ST elevation myocardial infarction demonstrated higher morbidity and mortality compared to those with non ST elevation myocardial infarction. In similar studies, Mann et al. [51] reported that prolonged QT was strongly and independently associated with mortality among patients with STEMI in their six-year study on 4936 patients. In addition, QT values may help identify high-risk patients and determine which ones should be followed up longer.

Gadelata et al. [52] indicated that QTc prolongation (>458 ms) observed on admission electrocardiography serves as an independent predictor of mortality risk in NSTEMI patients. Jiménez-Candil et al. [53] indicated an elevated risk of mortality, recurrent ischemia, or necessity for urgent revascularization at 17 months of follow-up in non ST elevation myocardial infarction patients with a QTc exceeding 450 ms upon admission. Demirtas et al. [54] found that QTc prolongation occurred more frequently in NSTEMI patients compared to those with unstable angina. This was linked to an increased occurrence of short-term adverse effects. Akgümüş et al. [55] observed a markedly elevated QTc in patients with three-vessel disease compared to those with one-vessel illness. QTc prolongation is a crucial clinical instrument for the early diagnosis of ischemia and the identification of patients at elevated risk for malignant arrhythmias [56]. Gadaleta et al. [52] indicated that QTc prolongation elevated the risk of cardiovascular events by 19 times in patients with non ST elevation myocardial infarction who exhibited neither normal electrocardiography findings nor new ischemic alterations at presentation. In NSTEMI patients with a QTc exceeding 0.458 ms, the incidence of composite outcomes including mortality, acute myocardial infarction, and percutaneous or surgical revascularization was higher 30 days post hospital discharge compared to individuals without cardiovascular events. Our investigation revealed a sensitivity of 99.7% and a specificity of 99.2% in forecasting mortality in patients with extended QTc (AUC: 0.975, 95% CI: 0.954–0.996). A significant connection between QTc prolongation and mortality was identified. A regression study revealed that QTc prolongation may serve as a predictor parameter for 30-day and 180-day mortality in both univariate and multivariable models. We suspect that QTc prolongation, when associated with alterations in lead aVR, may indicate a more prognostically severe acute coronary syndrome.

*Prognostic significance of QTc alterations in lead aVR:* The severity of coronary artery disease correlates with the myocardial area at risk, elevated plaque burden, extent of coronary stenosis, and multivessel disease. Moreover, vasospasm resulting from this also influences perfusion deficits and the magnitude of ischemic regions. Elevated QTc values after acute ischemia correlate with heightened severity of coronary disease [57]. Tracking the variations in QTc over time is considered a promising indicator for forecasting the heightened risk of acute coronary syndrome [18]. In the acute coronary syndrome cohort, the presence of aVR-STE is associated with a higher prevalence of three-vessel disease, with the likelihood of increasing proportionally to the degree of elevation. Likewise, the presence of aVR-STD correlates with an increased likelihood of myocardial infarction. Consequently, alterations in the aVR lead serve as an independent prognostic indicator of outcomes and death in acute coronary syndrome [58]. Based on the results, alterations in aVR derivation and QTc prolongation are significant risk factors. It is important to highlight that a shift in aVR derivation, when coupled with QTc prolongation in acute coronary syndrome, significantly elevates the risk. The primary findings of the study are as follows: (a) a broad ischemic area in acute coronary syndrome correlates with increased occurrences of ischemic heart failure, malignant arrhythmias, cardiac tamponade, and acute pulmonary edema in patients; (b) QTc and its distribution are significantly elevated in ASC-related derivations; (c) prolonged QTc with alterations in aVR derivation is markedly higher in predicting 30-day and 180-day mortality compared to the general population; (d) the association between aVR and QTc may serve as a crucial predictive parameter for both two-vessel and three-vessel disease, as well as potential complications and mortality. If alterations in the aVR derivation coincide with QTc prolongation, we believe this may indicate a higher mortality risk both during hospitalization and in the long term, necessitating prompt coronary angiography.

*Limitations:* The present research has several limitations. The analysis pertains to a retrospective observational study. Secondly, despite the substantial sample size, challenges arose in follow-up and accessibility. The subgroups of the STEMI patient cohort were not assessed. The elevated mortality rate may be attributed to the inclusion of high-risk unstable angina, ST elevation myocardial infarction, and NSTEMI patients in the cohort. If we had included stable angina cases, the mortality and complication rates would have been relatively lower. Fourth, we were unable to incorporate medicines that influence both aVR and QTc. Patients with COVID-19 and individuals with positive PCR tests were excluded from the trial, although they may have been exposed throughout the follow-up period. Upon arrival at the emergency department, patients were admitted to the coronary intensive care unit or ward within 30 min following the diagnosis of acute coronary syndrome. Treatment was conducted by the European Society of Cardiology guidelines in both the emergency department and cardiology, therefore excluding the consideration of other therapeutic modalities. Furthermore, substances that could extend QTc, electrolyte disturbances, acidosis, congenital long QT syndrome, and similar factors were systematically excluded from the study. Nevertheless, there may be instances that were neglected or emerged subsequently. Additionally, aortic stenosis was not considered. This could be a potential constraint. Finally, our study population included only patients who underwent coronary angiography because we aimed to determine the relationship between electrocardiography findings and angiographic findings. This may limit the generalizability of our findings to a larger population presenting with acute coronary syndrome.

## 5. Conclusions

Our research indicated that QTc prolongation is an independent factor influencing cardiovascular risk in patients with acute coronary syndrome exhibiting aVR changes on electrocardiography. The QTc, easily calculable through straightforward methods, may pose a significant risk factor when threshold values exceed 483 ms in ST elevation myocardial infarction, 475 ms in non ST elevation myocardial infarction, and 506 ms in patients with mortality in aVR-STE. Moreover, in aVR-STD, the cut-off values of 465 ms for STEMI, 457 ms for NSTEMI, and 509 ms for those with mortality should be considered. Should alterations in lead aVR coincide with QTc prolongation, it may indicate a compromised tricuspid valve and a bad prognosis, warranting the consideration of angioplasty in the near term. Consequently, patients with acute coronary syndrome, whether in the emergency department or coronary units, can gain insight into their prognosis with this straightforward application. Nonetheless, we recognize that additional research is necessary to validate and compare the prognostic significance of alterations in aVR derivation and the extension of the QTc.

## Figures and Tables

**Table 1:** Analysis of acute coronary syndrome basic characteristics and laboratory results according to aVR groups.

aVR Derivation					
		All Patients Mean (SD) n:1273 (100%)	ST Elevation Mean (SD) n:696 (54.7%)	ST Depression Mean (SD) n:577 (45.3%)	*p*-Value
Age, year		63.23 (10.06)	63.77 (10.10)	62.58 (9.98)	0.030
Gender	Female	548 (43)	296 (42.5)	252 (43.7)	0.691
	Male	725 (57)	400 (57.5)	325 (56.3)	
Laboratory	Glucose, mg/dL	132.01 (38.87)	139.55 (41.83)	122.93 (32.78)	<0.001
	Troponin I, pg/dL	1.41 (1.89)	1.60 (2.06)	1.19 (1.63)	0.001
Comorbidity	Diabetes Mellitus	561 (44.1)	351 (62.6)	210 (37.4)	<0.001
	Hypertension	859 (67.5)	469 (54.6)	390 (45.4)	0.952
	Tobacco/Cigarette	633 (49.7)	357 (56.4)	276 (43.6)	0.237
Acute Coronary Syndrome	Unstable Angina	622 (48.9)	307 (24.1)	315 (24.7)	0.005
	STEMI	409 (32.1)	250 (19.6)	159 (12.5)	
	NSTEMI	242 (19.0)	139 (10.9)	103 (8.1)	
Two- and Three-Vessel Disease	No	881 (69.2)	435 (34.2)	446 (35.0)	<0.001
	Two-Vessel Disease	234 (18.4)	131 (10.3)	103 (8.1)	
	Three-Vessel Disease	158 (12.4)	130 (10.2)	28 (2.2)	
Complication	No	833 (65.4)	433(34.0)	400 (31.4)	0.001
	Ischemic Heart Failure	295 (23.2)	160 (12.6)	135 (10.6)	
	Ventricular Tachycardia	48 (3.8)	35 (2.7)	13 (1.0)	
	AV Block	44 (3.5)	28 (2.2)	16 (1.3)	
	Cardiac Tamponade	19 (1.5)	15 (1.2)	4 (0.3)	
	Pulmonary Edema	34 (2.7)	25 (2.0)	9 (0.7)	
Mortality (30 days)	No	1144 (89.9)	593 (46.6)	551 (43.3)	<0.001
	Yes	129 (10.1)	103 (8.1)	26 (2.0)	
Mortality (180 days)	No	1092 (85.8)	559 (43.9)	533 (41.9)	<0.001
	Yes	181 (14.2)	137 (10.8)	44 (3.5)	

SD: standard deviation, STEMI: ST elevation myocardial infarction, NSTEMI: non ST elevation myocardial infarction, AV: atrioventricular, *p*: statistical significance (<0.05).

**Table 2:** Relationship between QTc, ST elevation in lead aVR, and depression status with acute coronary syndrome and mortality.

QTc					
aVR Derivation			Mean	95% Cl	*p*-Value
Elevation	Acute Coronary Syndrome Group	Unstable Angina	406.37	401.88–410.86	<0.001
		STEMI	483.31	479.08–487.54	
		NSTEMI	474.98	470.58–479.38	
	Mortality	No	437.65	433.72–441.58	<0.001
		Yes	505.60	503.69–507.51	
Depression	Acute Coronary Syndrome Group	Unstable Angina	385.55	381.03–390.08	<0.001
		STEMI	465.10	458.42–471.78	
		NSTEMI	457.52	449.80–564.25	
	Mortality	No	416.15	411.64–420.66	<0.001
		Yes	508.73	506.83–510.63	

STEMI: ST elevation myocardial infarction, NSTEMI: non ST elevation myocardial infarction, *p*: statistical significance (<0.05).

**Table 3:** Correlation analysis of aVR derivation and mortality with variables.

aVR Derivation and Mortality	aVR Derivation		Mortality	
	r	*p*	r	*p*
Age, year	−0.061	0.030	0.075	0.007
Glucose, mg/dL	−0.190	<0.001	0.132	<0.001
Corrected QT interval (QTc), ms	−0.280	<0.001	0.505	<0.001
TroponinI, ng/dL	−0.096	0.001	0.254	<0.001

r: Correlation coefficient, *p*: statistical significance (<0.05).

**Table 4:** Regression analysis of aVR derivation and mortality with variables.

aVR Derivation and Mortality		Univariate			Multivariate		
		OR	95% Cl	*p*	OR	95% Cl	*p*
aVR Derivation	Age, year	0.829	0.997–0.999	0.036	0.829	0.996–1.010	0.743
	Glucose, mg/dL	0.988	0.985–0.991	<0.001	0.991	0.988–0.998	0.006
	QTc, ms	0.991	0.989–0.993	<0.001	0.997	0.988–0.993	<0.001
	TroponinI, ng/dL	0.887	0.834–0.943	<0.001	1.088	1.014–1.182	0.020
Mortality	Age, year	1.025	1.006–1.044	0.009	0.995	0.991–1.030	0.768
	Glucose, mg/dL	1.011	1.006–1.015	<0.001	1.006	0.996–1.020	0.182
	QTc, ms	1.300	1.239–1.365	<0.001	1.302	1.240–1.368	<0.001
	TroponinI, ng/dL	1.359	1.255–1.471	<0.001	1.035	0.866–1.240	0.697

OR: odds ratio,95% CI: confidence interval, QTc: corrected QT interval, *p*: statistical significance (<0.05).

## Data Availability

All data are available on request without restriction.
